# Clinical Features and Management of Women with Borderline Ovarian Tumors in a Single Center in Brazil

**DOI:** 10.1055/s-0039-1683415

**Published:** 2019-03

**Authors:** Adriana Yoshida, Bárbara Virginia Gonçalves Tavares, Luís Otavio Sarian, Liliana Aparecida Lucci Ângelo Andrade, Sophie Françoise Derchain

**Affiliations:** 1 Department of Obstetrics and Gynecology, Faculty of Medical Sciences, Universidade Estadual de Campinas, Campinas, SP, Brazil; 2 Department of Pathology, Universidade Estadual de Campinas, Campinas, SP, Brazil

**Keywords:** borderline ovarian tumor (BOT), treatment, fertility sparing surgery, recurrence, tumores borderline de ovário (TBO), tratamento, cirurgia preservadora de fertilidade, recorrência

## Abstract

**Objective** The aim of the present study was to describe and analyze data of 57 women with borderline ovarian tumors (BOTs) regarding histological characteristics, clinical features and treatment management at the Department of Obstetrics and Gynecology of the Universidade Estadual de Campinas (Unicamp, in the Portuguese acronym).

**Methods** The present retrospective study analyzed data obtained from clinical and histopathological reports of women with BOTs treated in a single cancer center between 2010 and 2018.

**Results** A total of 57 women were included, with a mean age of 48.42 years old (15.43–80.77), of which 30 (52.63%) were postmenopausal, and 18 (31.58%) were < 40 years old. All of the women underwent surgery. A total of 37 women (64.91%) were submitted to complete surgical staging for BOT, and none (0/57) were submitted to pelvic or paraortic lymphadenectomy. Chemotherapy was administered for two patients who recurred. The final histological diagnoses were: serous in 20 (35.09%) cases, mucinous in 26 (45.61%), seromucinous in 10 (17.54%), and endometrioid in 1 (1.75%) case. Intraoperative analyses of frozen sections were obtained in 42 (73.68%) women, of which 28 (66.67%) matched with the final diagnosis. The mean follow-up was of 42.79 months (range: 2.03–104.87 months). Regarding the current status of the women, 45 (78.95%) are alive without disease, 2 (3.51%) are alive with disease, 9 (15.79%) had their last follow-up visit > 1 year before the performance of the present study but are alive, and 1 patient (1.75%) died of another cause.

**Conclusion** Women in the present study were treated according to the current guidelines and only two patients recurred.

## Abstract (pt)

**Resumo:**

**Objetivo** O objetivo do presente estudo foi descrever uma série de 57 mulheres com tumores borderline de ovário (TBO) em relação às características histológicas, clínicas, e ao manejo do tratamento realizado no Departamento de Obstetrícia e Ginecologia da Universidade Estadual de Campinas (Unicamp).

**Métodos** O presente estudo retrospectivo analisou dados obtidos dos registros clínicos e histopatológicos de mulheres com TBO tratadas em um único centro oncológico de 2010 a 2018.

**Resultados** Um total de 57 mulheres foram incluídas, com uma média de idade de 48,42 anos (15,43–80,77), das quais 30 (52,63%) eram menopausadas, e 18 (31,58%) tinham < 40 anos. Todas as mulheres foram operadas. Um total de 37 mulheres (64,91%) foram submetidas a cirurgia de estadiamento completo para TBO, e nenhuma foi submetida a linfadenectomia pélvica ou paraórtica. O tratamento com quimioterapia foi administrado em duas pacientes que recidivaram. Os diagnósticos histológicos finais foram: seroso em 20 mulheres (35,09%), mucinoso em 26 (45,61%), seromucinoso em 10 (17,54%) e endometrióide em 1 (1,75%). A avaliação histológica intraoperatória foi realizada em 42 (73,68%) das mulheres, das quais 28 (66,67%) foram compatíveis com os diagnósticos finais. O tempo médio de seguimento foi de 42,79 meses (variando de 2,03 a 104,87 meses). Em relação ao status atual das mulheres, 45 (78.95%) estão vivas sem doença, 2 (3,51%) estão vivas com doença, 9 (15.79%) tiveram a última consulta de seguimento há > 1 ano antes da realização do presente estudo, mas estão vivas, e 1 paciente faleceu por outra causa.

**Conclusão** As mulheres do presente estudo foram tratadas de acordo com as recomendações atuais e apenas duas mulheres apresentaram recorrência.

## Introduction

Borderline ovarian tumor (BOT) is a peculiar type of tumor with an indolent behavior. This disease occurs in women 10 years younger than those with epithelial ovarian cancer (EOC). They are more frequently diagnosed in the earlier stages (75% in stage I), leading to an excellent prognosis. The treatment is based on the surgical removal of the disease with emphasis on fertility-sparing surgery in women of childbearing age.^1^ ^2^

Surgery is indicated for women with suspicious adnexal masses detected in ultrasound (US) or in other preoperative exams. The preoperative differential diagnosis is important, allowing adequate surgery planning; however, performances of imaging and biomarkers may fail to discriminate EOC in the initial stages from BOT cases.^3^ ^4^ Besides, frozen-section readings will never achieve 100% of accuracy; however, despite the possibility of underdiagnosis and overdiagnosis, they may provide guidance in most cases.^2^ ^5^ ^6^

A careful inspection of the abdominal and pelvic cavities should be performed for adequate staging and complete removal of the disease. Surgical staging generally includes cytologic washings, resection of the tumor, infracolic omentectomy and peritoneal biopsies. Routine lymphadenectomy is not recommended.^2^ For early stage (stage I), cautiously unilateral cystectomy or tumorectomy are considered treatment options if no surface involvement of the ovary is present.^7^ This conservative procedure is of major importance for premenopausal women who harbor bilateral disease or have a single ovary and desire childbearing.^8^ Although recurrences are more frequent when the ovary is preserved, a negative impact on survival is not observed, since most of the recurrences are of the borderline type and may be salvaged with surgical treatment.^1^ ^9^ Unilateral salpingo-oophorectomy (USO) is another option for patients with unilateral disease. For peri or postmenopausal women, total hysterectomy (TH) and bilateral salpingo-ophorectomy (BSO) are the treatment of choice.^8^

For advanced stages, the whole visible disease should be surgically removed, if feasible. Extraovarian disease (invasive and noninvasive implants) may be present, but even if peritoneal implants are present, a normal contralateral ovary may be preserved in young patients.^2^ Although there is no consensus, postoperative chemotherapy is recommended for those with serous BOT with invasive implants, in selected cases, since invasive peritoneal implants are equivalent to low-grade serous carcinoma.^2^ ^10^

The objective of the present study was to describe and analyze the clinical features, the tumor histology, and the treatment of women with BOT in a single cancer center, in Campinas, state of São Paulo, Brazil, between the years 2010 and 2018.

## Methods

This is a retrospective study, which evaluated women included from the period of January 2010 to April 2018 at the Centro de Atenção Integral à Saúde da Mulher (CAISM, in the Portuguese acronym) of the Universidade Estadual de Campinas (Unicamp, in the Portuguese acronym), a tertiary cancer center specialized in gynecological malignancies. The University Research Ethics Committee (number 1092/2009) approved the present study. The inclusion criteria were women referred to the pelvic oncology clinic due to adnexal masses detected in US or in other imaging exams. Patients were consecutively included after signing a consent form, and were submitted to a physical exam, and a pelvic US was scheduled. Blood samples were collected for serum tumor marker (CA125) dosage. Surgical procedures were scheduled after the evaluation of the pelvic US, of the CA125 level, and of the physical exam. In the present study, we have enrolled 1,481 consecutive women, and we have excluded those with no surgical indication (706 women). Among those who were submitted to surgery or biopsy, we chose those with BOT histology. In total, 57 women with ovarian tumors were included. Tissue specimens were analyzed by pathologists specialized in gynecologic pathology, according to the guidelines of the World Health Organization (WHO) International Classification of Ovarian Tumors.^11^ For tumor staging, we followed the International Federation of Gynecologists and Obstetricians (FIGO) classification.^12^ Bilateral tumors were found in 12 women; for categorization purposes, the tumor with the worst prognosis was taken into account. Postmenopausal status was defined as > 1 year of amenorrhea or age ≥50 years in the case of previous hysterectomy. Fertility-sparing surgery was considered when sparing of the uterus, along with one or both ovaries, occurred during the surgical treatment. All of the clinical, histological and surgical data were obtained from the records of the patients. Complementary information about survival for women whose last follow-up visit were >1 year before the performance of the present study were obtained through the internet site of National Registry of individuals in November, 2018.^13^

### CA125 Measurement

We have collected blood samples from patients by peripheral vein puncture before surgery or percutaneous biopsy, and the samples were stored in serum separator tubes, for CA125 dosage. Serum CA125 was determined by the CA125 II test, through the electrochemiluminescence technique in the automatic analyzer Cobas e411 (Roche Diagnostics GmbH, Mannheim, Germany) according to the instructions of the manufacturer.

### Statistical Analysis

Data were analyzed using the R Environment for Statistical Computing Software (R Foundation for Statistical Computing, Vienna, Austria).^14^ We determined the mean values (with corresponding range) for age, age of menarche, body mass index (BMI), serum CA125 levels, and follow-up.

## Results

Table 1 shows the demographic characteristics of the patients with BOT. The mean age of the women included was 48.42 years old (15.43–80.77). The mean age at menarche was 13 years old (10–16), and the mean BMI was 28.11 kg/m^2^. Approximately half of the group was postmenopausal (47.37%), and the majority of women presented stage I disease (82.46%). The predominant histologies were serous and mucinous. Most of the women (73.68%) were submitted to frozen section of the tumor. Results of the intraoperative histological analysis were: 9 benign (21.43%) (1 seromucinous and 8 mucinous BOT), 28 BOT (66.67%), 5 malignant (11.9%) (2 serous and 3 mucinous BOT) (data not shown in table). Most of the women underwent nonconservative surgery, and laparotomy was the most preferred approach.

**Table 1:** Demographics of women with borderline ovarian tumors

*Characteristics*	
Mean age (range), years old	48.42 (15.43–80.77)
Mean age at menarche (range), years old	13 (10–16)
Mean body mass index (range), kg/m^2^	28.11 (17.69–41.03)
*Age groups*	*n* (%)
< 50 years old	28 (49.12)
≥50 years old	29 (50.88)
*Menopause*	
No	30 (52.63)
Yes	27 (47.37)
*FIGO stage*	
I	47 (82.46)
II	3 (5.26)
III	7 (12.28)
IV	0
*Histology*	
Serous	20 (35.09)
Mucinous (intestinal type)	26 (45.61)
Seromucinous	10 (17.54)
Endometrioid	1 (1.75)
*Frozen section*	
No	15 (26.32)
Yes	42 (73.68)
*Hysterectomy*	
No	15 (26.32)
Yes	42 (73.68)*
*Ovarian preservation*	
No	44 (77.19)
Yes	13 (22.81)
*Surgical procedure by*	
Laparotomy	51 (89.47)
Laparoscopy	6 (10.53)

Abbreviations: FIGO, International Federation of Gynecology and Obstetrics.

* Three patients had previous hysterectomy

Table 2 shows the clinical features of the patients, the histological descriptions of their tumors, and information about the follow-up, according to the tumor subtype. All of the patients with mucinous BOT presented disease in stage I. The mean serum level of CA125 was > 35 U/ml for all subtypes. CA125 levels were missing for 2 women (1 with serous and the other with mucinous BOT). Microinvasion was presented in 10 women, and 10 women presented with implants (2 women presented with invasive implants). Two patients had serous BOT with micropapilary pattern. Three women had neoplastic cells in ascites, 2 had tumor rupture before or during the surgery, and 11 had involved surface tumor (no involvement in mucinous subtype). Two patients with mucinous BOT presented intraepithelial carcinoma. A total of 45 women (78.95%) are alive without disease, 2 patients (3.51%) are alive with disease, 1 patient (1.75%) died after a femur fracture (she was without disease), and 9 women (15.79%) had their last follow-up visit > 1 year before the performance of the present study, but are alive according to data obtained through the National Registry of individuals in November 2018, as already explained in the Methods section.

**Table 2:** Clinical and tumor characteristics according to the histological subtypes of borderline ovarian tumors

	BOT histological subtypes			
Clinical characteristics, *n* (%)	Serous (*n* = 20)	Mucinous (intestinal type*,* *n* = 26)	Seromucinous (*n* = 10)	Endometrioid (*n* = 1)
Stage				
I	14 (70)	26 (100)	6 (60)	1 (100)
II	2 (10)	0	1 (10)	0
III	4 (20)	0	3 (30)	0
Mean serum CA125 (range) U/ml	340.88* (14–2,680)	68.38* (7.24–395.7)	287.65 (13.82–695.9)	46.25
Tumor characteristics, n (% in relation to n)				
Bilateral tumor	7 (35)	1 (3.85)	4 (40)	0
Microinvasion	4 (20)	1 (3.85)	5 (50)	0
Invasive implants	1 (5)	0	1 (10)	0
Noninvasive implants	5 (25)	0	3 (30)	0
Without implants	14 (70)	26 (100)	6 (60)	1 (100)
Micropapillary pattern	2 (10)	0	0	0
Neoplastic cell in ascites	2 (10)	0	1 (10)	0
Tumor rupture before or during surgery	0	1 (3.85)	1 (10)	0
Involved surface of tumor	7 (35)	0	4 (40)	0
with intraepithelial carcinoma	0	2 (7.69)	0	0
Patient status, n (% in relation to n)				
Alive without disease	19 (95)	19 (73.08)	6 (60)	1 (100)
Alive with disease	0	1 (3.85)	1 (10)	0
Alive (last follow-up visit > 1 year ago)**	1 (5)	5 (19.23)	3 (30)	0
Dead (other cause)	0	1 (3.85)	0	0

Abbreviation: BOT, borderline ovarian tumor.

* missing dosage for one patient;

** living status ascertained through the National Registry of individuals (CPF, in the Portuguese acronym)

Table 3 shows the types of surgical procedures performed and follow-up data according to the stage of the disease, menopausal status, and age. Thirteen women were submitted to ovary conservative surgery, the great majority of which had both ovaries resected, although bilateral disease was seen in only 12 women. Complementary information to table 3: 1) 1 patient who was submitted to TH + USO had only one ovary; 2) all of the 13 women who were submitted to USO had both ovaries before surgery and, among them, 1 woman had been previously submitted to hysterectomy; 3) 37 women (64.91%) were submitted to complete surgical staging for BOT, which included cytologic washings, resection of the ovarian tumor, infracolic omentectomy, pelvic and abdominal peritoneum biopsies. The other 20 (35.09%) were not submitted to a reoperation for staging procedures, since in the first surgery no macroscopic disease was left after performing a careful inspection of the abdominal and pelvic cavities. No women (0/57) underwent pelvic or paraortic lymphadenectomy; 4) 18 women were < 40 years old [7 had TH + BSO, 10 USO and 1 USO + Contralateral Cystectomy (CC)]; 5) among the postmenopausal women, (26/27) 96% had both ovaries removed; 6) in the subgroup of 26 women with mucinous BOT, 12 underwent an appendectomy, without malignancy detected in the appendix; 7) the mean follow-up was of 42.79 months (range: 2.03–104.87 months).

**Table 3:** Type of surgical procedure and patient status according to stage, menopausal status, and age

	Stage			Menopausal status		Age		
Type of surgical procedure, *n* (%)	I	II	III	Premenopausal	Postmenopausal	< 40 years old	40–49 years old	≥50 years old
Total hysterectomy with bilateral salpingoophorectomy	31 (65.96)	2 (66.67)	5 (71.43)	16 (53.33)	22 (81.48)	7 (38.89)	8 (80)	23 (79.31)
Bilateral salpingoophorectomy	4 (8.51)	0	0	0	4 (14.81)	0	0	4 (13.79)
Total hysterectomy with unilateral salpingoophorectomy	0	0	1 (14.28)*	1 (3.33)*	0	0	0	1* (3.45)
Unilateral salpingoophorectomy	12 (25.53)**	1 (33.33)**	0	12 (40)	1 (3.70)	10 (55.55)	2 (20)	1 (3.45)
Unilateral salpingoophorectomy + contralateral cystectomy	0	0	1 (14.28)	1 (3.33)	0	1 (5.55)	0	0
*Patient status, n (%)*								
Alive without disease	38 (80.85)	2 (66.67)	5 (71.43)	26 (86.67)	19 (70.37)	15 (83.33)	9 (90)	21 (72.41)
Alive with disease	1 (2.13)	0	1 (14.28)	1 (3.33)	1 (3.70)	0	0	2 (6.90)
Alive (last follow-up visit >1 year ago)***	7 (14.89)	1 (33.33)	1 (14.28)	3 (10)	6 (22.22)	3 (16.67)	1 (10)	5 (17.24)
Dead (other cause)	1 (2.13)	0	0	0	1 (3.70)	0	0	1 (3.45)
Total no. of patients, n (%)	47 (100)	3 (100)	7 (100)	30 (100)	27 (100)	18 (100)	10 (100)	29 (100)

* patient with previous oophorectomy.

** no patient with previous oophorectomy, one patient < 50 years old with previous hysterectomy.

*** living status ascertained through the National Registry of living individuals (CPF, in the Portuguese acronym).

Out of the 57 patients, 2 recurred: 1 of the patients, who is alive with disease, was submitted to the 1^st^ surgery in 2011 (she was 52 years old at the time) with TH and BSO, infracolic omentectomy, and appendectomy. Peritoneal lavage was collected for analysis. Noninvasive implants were presented in the omentum, in the uterus, in the appendix, or in the fallopian tubes. The histological diagnosis was seromucinous BOT stage IIIb. In 2013, noninvasive implants were resected in the pelvis, and in 2015 she presented with recurrence of the disease with invasive implants in the pelvic peritoneum, in the cecum, and in the celiac artery, as well as noninvasive implants in the small intestine. A complete debulking surgery was not achieved, and in 2018 she received chemotherapy with carboplatin and taxol for disease progression, and after 5 cycles without response, oral cyclophosphamide was initiated and is still maintained until November 2018, with stable disease.

Another patient, 68 years old, was first admitted to the hospital and submitted to surgery in 2013. She was submitted to TH + BSO, appendectomy, infracolic omentectomy, multiple peritoneum biopsies, and the final diagnosis was mucinous BOT stage Ia (intestinal type, without microinvasion nor intraepithelial carcinoma). In 2018, she presented with recurrence in the lungs. The pleural biopsy revealed adenocarcinoma metastatic from the ovaries. She is being treated with chemotherapy with carboplatin and taxol, 4 cycles were administered until November 2018.

## Discussion

In the present study of women with BOT treated in a single center, we have found that 56 out of 57 patients are alive, reflecting the good prognosis of this disease. Although more time of follow-up is needed to detect recurrences, we could observe an indolent behavior of this tumor, as has already been described in the current literature.^15^ ^16^

In our casuistic, among 18 women < 40 years old, 11 underwent fertility-sparing surgery [10 were submitted to USO, and 1 to USO + Contralateral Cystectomy (CC)], and 7 were submitted to TH + BSO (4 had stage III disease and 3 had stage I; all of them were > 35 years old, with at least 1 child each; furthermore, 6 had bilateral tumors, and 1 had unilateral disease). A meta-analysis with focus on recurrence risk included 2,752 women with BOT who underwent conservative surgery: 817 were submitted to cystectomy, 89 to bilateral cystectomy, 1,686 to USO, and 118 to USO and Contralateral Cystectomy (CC). Of the patients who underwent cystectomy, bilateral cystectomy, USO, and USO + CC, the pooled recurrence estimates were of 25.3%, 25.6%, 12.5% and 26.1%, respectively. The authors concluded that cystectomy in unilateral serous BOT was significantly associated with a higher recurrence rate, although no impact on survival could be demonstrated.^17^

Another recent study included 132 women, of which 112 (85%) underwent a fertility-sparing procedure, and 60 (46%) had one involved ovary preserved. Fifty patients (24%) presented recurrences. Fertility preservation (hazard ratio [HR] = 2.57; 95% confidence interval (CI): 1.1–6*;* *p* = 0.029) and advanced stage (HR = 4.15; 95% CI: 2.3–7.6; *p *< 0.001) were independently associated with recurrence on a multivariate analysis, although fertility preservation was not associated with compromised overall survival.^9^

Moreover, in a recent study involving 4,943 women, surgical staging patterns for hysterectomy and lymphadenectomy were not associated with survival of women with stage I BOT. According to the study, both procedures could be omitted in the surgical management of stage I BOT, particularly for stage Ia disease, regardless of the age of the patient.^18^ Staging in case of BOT is a controversial issue, especially for the mucinous subtype. De Decker et al^19^ included 74 mucinous BOT patients in their study. Forty-six (62.2%) underwent a staging procedure. In 12 (26.1%) patients, only omental tissue was obtained, in 32 (69.6%) patients, omental and peritoneal biopsies were obtained, and in 2 (4.3%) patients, only peritoneal biopsies were obtained. No implants were seen upon microscopic examination in any of the patients. Only 2 patients (3%) developed a recurrence. The study suggests that since no extraovarian disease was found, staging procedures in the case of mucinous BOT could be omitted.^19^ In the present study, 20 women (35.09%) remained as presumed stage I BOT, as they were not submitted to omental nor peritoneal biopsies (11 mucinous, 5 serous, 3 seromucinous, and 1 endometrioid BOT), and there was no recurrence among them.

In our casuistic, 12 patients (46.15%) among a total of 26 with mucinous BOT were submitted to appendectomy, all of them without neoplasia in the postoperative histologic diagnosis of the appendix. Surgical guidelines recommend this procedure as part of the staging and treatment of mucinous ovarian neoplasms, since it might help to: 1) exclude a primary appendix origin of the ovarian tumor, and 2) perform the debulking of the disease.^20^ In a recent systematic review, appendix involvement in mucinous BOTs appears to be extremely rare, and microscopic appendix involvement is highly unlikely in apparently normal appendixes. Therefore, in the case of normal appearance of this organ at the time of the primary surgery, appendectomy is not mandatory. Moreover, a patient with an appendix with normal appearance during the primary surgery with postoperative diagnosis of mucinous BOT does not need to be submitted to a second look intervention for this procedure.^20^

More than 96% of BOTs are of the serous or mucinous subtypes. Other rare types are endometrioid, clear cell, or Brenner (transitional cell) BOTs.^21^ This prevalence of subtypes was also observed in our cases. There are still some controversies about prognostic factors for recurrences related to tumor characteristics or patient factors. Uzan et al,^22^ in a large series of stage I BOT (total of 254 patients), including119 patients treated conservatively, showed that after a median follow-up of 45 months, 43 patients had recurred. In the subgroup of conservatively treated patients (75% of their population), the risks of recurrences were increased in patients with serous BOT, in those who had undergone a cystectomy, in patients with stage IB disease, and in those with micropapillary pattern. Besides, mucinous BOT and the presence of a micropapillary pattern were identified as prognostic factors for invasive disease.^22^ In our casuistic, we had 2 patients with recurrences; both were ≥50 years old, presented unilateral disease, and underwent nonconservative surgery. May et al,^16^ in a study involving 275 patients with BOT, found that advanced stage was the most important prognostic factor. Also, elevated preoperative serum CA125 and the presence of micropapillary features correlated with advanced stage at presentation. According to this study, recurrent disease is rare in optimally staged, completely resected, early stage BOT, without these high-risk features.^16^

## Conclusion

In conclusion, since BOTs have an indolent behavior, it is important to consider fertility-sparing surgery in patients who desire childbearing. Lymphadenectomy for all subtypes and routine appendectomy for mucinous BOT are not reccomended. Chemotherapy for recurrences, such as invasive disease, is still controversial. Finally, women in the present study were treated according to the current guidelines, and only two patients recurred.
